# Course and prognosis of recovery for chronic non-specific low back pain: design, therapy program and baseline data of a prospective cohort study

**DOI:** 10.1186/1471-2474-12-252

**Published:** 2011-11-02

**Authors:** Karin Verkerk, Pim AJ Luijsterburg, Inge Ronchetti, Harald S Miedema, Annelies Pool-Goudzwaard, Jan-Paul van Wingerden, Bart W Koes

**Affiliations:** 1 Rotterdam University of Applied Sciences, Rotterdam, The Netherlands; 2 Spine & Joint Centre, Rotterdam, Netherlands; 3 Department of General Practice, Erasmus MC, University Medical Center, Rotterdam, Netherlands; 4 Department of Neuroscience, Erasmus MC, University Medical Center, Rotterdam, Netherlands

## Abstract

**Background:**

There has been increasing focus on factors predicting the development of chronic musculoskeletal disorders. For patients already experiencing chronic non-specific low back pain it is also relevant to investigate which prognostic factors predict recovery. We present the design of a cohort study that aims to determine the course and prognostic factors for recovery in patients with chronic non-specific low back pain.

**Methods/Design:**

All participating patients were recruited (Jan 2003-Dec 2008) from the same rehabilitation centre and were evaluated by means of (postal) questionnaires and physical examinations at baseline, during the 2-month therapy program, and at 5 and 12 months after start of therapy. The therapy protocol at the rehabilitation centre used a bio-psychosocial approach to stimulate patients to adopt adequate (movement) behaviour aimed at physical and functional recovery. The program is part of regular care and consists of 16 sessions of 3 hours each, over an 8-week period (in total 48 hours), followed by a 3-month self-management program. The primary outcomes are low back pain intensity, disability, quality of life, patient's global perceived effect of recovery, and participation in work. Baseline characteristics include information on socio-demographics, low back pain, employment status, and additional clinical items status such as fatigue, duration of activities, and fear of kinesiophobia. Prognostic variables are determined for recovery at short-term (5 months) and long-term (12 months) follow-up after start of therapy.

**Discussion:**

In a routine clinical setting it is important to provide patients suffering from chronic non-specific low back pain with adequate information about the prognosis of their complaint.

## Background

In the Netherlands, the annual incidence of back pain in the general population is estimated at 10-15% [1]. In 1999, chronic non-specific low back pain (CNLBP) was reported by 16.0% of Dutch working men, by 23.1% of non-working men, by 17.9% of working women and 27.4% of non-working women [2]. CNLBP has consequences for daily activity, use of health care services and ability to work. Most people with acute low back pain recover from their pain and/or disability and return to work within a few weeks [3]. Up to 3 months the self-limiting condition improves at a slower pace compared to the first month of recovery, and after 3 months the chance of recovery diminishes for patients with CNLBP [1,3-5]. However, CNLBP can fluctuate over time with (frequent) recurrences or exacerbations [6,7]. Identifying the factors that predict the prognosis of CNLBP can help physicians in the management of patients with CNLBP. Prognostic factors are suspected to differ between acute and chronic non-specific low back pain since the course of these two conditions differs [4,8]. The transition from acute non-specific low back pain to CNLBP has been well investigated [9-12], whereas studies on prognostic factors for recovery from CNLBP are scarce.

A recent systematic review investigating which outcome measurements were used to define recovery of low back pain in the past 10 years, concluded that almost every study defined recovery differently [13]. Although pain and disability were the outcome measurements most often used for defining recovery, a broader perspective may provide a more comprehensive health profile of the patient [14-16].

Therefore, we present the design of a cohort study that investigates the course of patients with CNLBP undergoing treatment in an outpatient rehabilitation centre. Also investigated are prognostic factors for recovery using the outcomes low back pain intensity, low back pain specific disability, generic health status, patient's global perceived effect of recovery and work participation on both the short (5 month) and long (12 month) term.

## Methods/Design

### Design

This study is a prospective cohort study. Patients were recruited (from January 2003 - December 2008) in a multidisciplinary outpatient rehabilitation clinic the 'Spine & Joint Centre' (SJC) in Rotterdam. The Medical Ethics Committee of SJC approved the study protocol and all participants provided informed consent.

### Participants

In the present study, low back pain is defined as 'non-specific low back pain', i.e. low back pain without a specified physical cause, such as nerve root compression (the radicular syndrome), trauma, infection or the presence of a tumour. Pain in the lumbosacral region is the most common symptom in patients with non-specific low back pain. Pain may also radiate to the gluteal region or to the thighs, or to both [17].

Patients with CNLBP (low back pain duration > 3 months) not recovering after primary and/or secondary care were referred by their general practitioner (GP) or specialist to the SJC for a diagnostic consultation.

The inclusion criteria for this study are:

• Men and women aged 18 years or over;

• Having CNLBP (i.e. a duration of low back pain for ≥ 3 months);

• Previous and insufficient treatment in primary and secondary care (e.g. physiotherapy);

• Signed informed consent.

Exclusion criteria are:

• Insufficient knowledge of the Dutch language;

• Signs indicating radiculopathy: asymmetric Achilles tendon reflex and/or (passive) straight leg raise test restricted by pain in the lower leg; positive magnetic resonance imaging findings for disc herniation;

• Recent (< 6 months) fracture, neoplasm or recent previous surgery (< 6 months) of the lumbar spine, the pelvic girdle, the hip joint, or the femur;

• Specific causes such ankylosing spondylitis and systemic disease of the locomotor system;

• Being pregnant or ≤ 6 months post-partum at the moment of consultation.

### Procedure in the SJC

**Figure 1:**
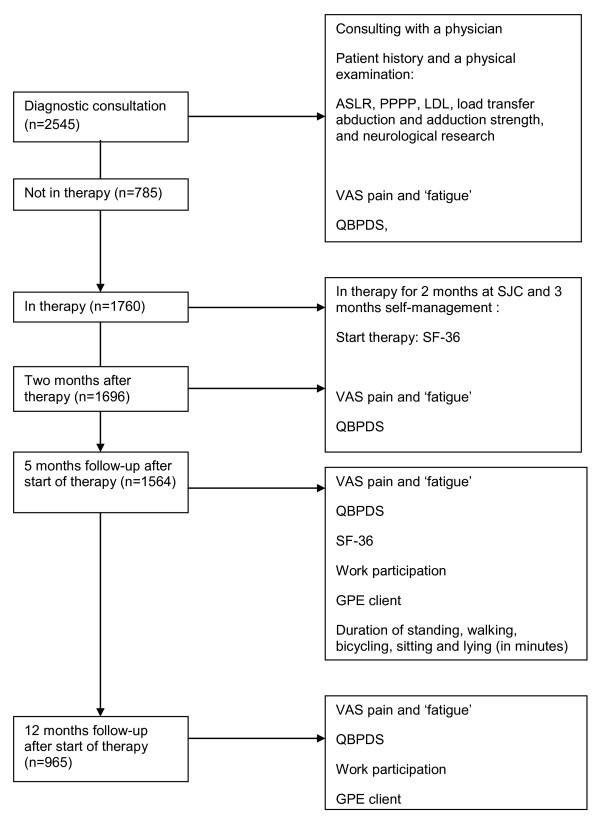
**Study design**. ASLR = Active Straight Leg Raise test; PPPP = Posterior Pelvic Pain Provocation test; LDL = longum dorsal sacroiliac ligament; VAS = Visual Analogue Scale; QBPDS = Quebec Back Pain Disability Scale; SF-36 = Short Form; TSK = Tampa Scale Kinesiophobia.

Based on a bio-psychosocial understanding of CNLBP the following steps are followed (Figure 1):

#### Intake (diagnostic consultation)

The intake is a 3-hour session in which: 1) the patient fills in psychometric questionnaires by computer; 2) a recording is made of the patient's strength (Isostation B200), a motion analysis of forward bending of the lumbar pelvic rhythm (video registration) of the trunk, and 3) the patients sees a physician for history taking and physical examination. The physician may request an additional consultation with a psychologist and/or manual physiotherapist before deciding on treatment management.

Patients meeting the inclusion criteria for the SJC are invited to participate in the multidisciplinary treatment program. Those not wishing to participate in this program are referred to their GP with a letter containing appropriate recommendations.

#### Therapy Program

In the therapy protocol, behavioural principles are applied to encourage patients to adopt adequate normal behavioural movement aimed at physical recovery. The program consists of 16 sessions of 3 hours each, over a 2-month period (a total of 48 hours) located in the SJC. During the program patients are educated to be self-supporting and to become 'their own therapist'. After this 2-month period, patients are encouraged to continue the training program independently for at least 3 months, twice a week, in a local, regular health centre located near their home environment. Five months after the start of the therapy program (2 months at SJC + 3 months self-supporting activity) the patient has a follow-up meeting.

#### 5-month follow-up after start of therapy

At the 5-month follow-up the patient fills in questionnaires, and discusses the recovery process with a focus on personal targets with regard to physical training, and psychological and social factors. A physical examination takes place and (if required) personal advice is provided by one of the therapists of the SJC.

#### 12-month follow-up after start of therapy

Via postal correspondence the patient is asked to fill in the 12-month questionnaires.

At the SJC a small group of patients follow treatment once a week for 4 months (instead of twice a week for 2 months). After the program is completed they are encouraged to continue their training program for at least 3 months in a regular health centre. At 7 and 14 months after start of therapy the same follow-up procedure is performed. The reason for the 'once a week' program is that some patients are unable to visit the SJC twice a week due to travelling and/or physical problems.

### SJC treatment program

**Figure 2:**
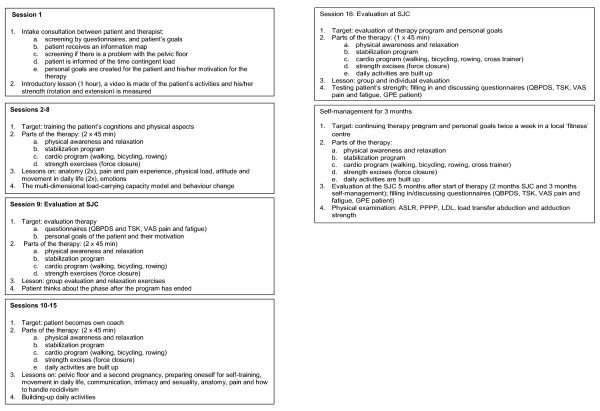
**Flow chart of therapy program**. VAS = Visual Analogue Scale; QBPDS **= Quebec Back Pain Disability Scale**; SF-36 = Short Form; TSK = Tampa Scale Kinesiophobia; GPE = Global Perceived Effect; ASLR = Active Straight Leg Raise; PPPP = Posterior Pelvic Pain Provocation test; LDL = Longum Dorsal sacroiliac Ligament.

Patients are treated in groups of 6 accompanied by 3 therapists. In the first session a personal treatment goal/plan is established with agreement from the patient. During the 9^th ^and 16^th ^sessions there is a 1:1 patient/therapist evaluation (in addition to the regular training program). The remainder of the treatment sessions consist of 1-hour training, a 1-hour group lesson, followed by another 1-hour training. The training consists of group training and/or individual coaching. Figure 2 presents the treatment protocol. The therapists (e.g., a physiotherapist, Mensendieck therapist, psychologist, health scientist, physician) are trained in the bio-psychosocial aspects of CNLBP.

The aim of the program is to normalise motion behaviour. This is done by modifying the patient's experience of movements and increasing the experienced quality of movements by learning about and training the reduction of compensatory mechanisms of a physical nature, e.g. increasing intra-abdominal pressure at low loads, breathing cessation during loading tasks, and extreme activity in all superficial muscles. During the program it is explained that the above-described compensatory mechanisms are present due to an interaction between biophysical and psychosocial factors (multidimensional) such as stress, psychological status and social factors. All these factors are treated by a multidisciplinary team.

The training starts to increase awareness of excessive tension of the muscles in trunk and extremities. The patient is stimulated to take breaks during daily activities by using tools like time contingent management and learning about his/her physical load and physical capacity [18,19]. Breathing techniques are used in combination with a stabilisation program to normalise the activity of the m. multifidus, m. transversus abdominis [20-25], diaphragm and pelvic floor (the 'inner tube system'). In a later stage different coordination patterns of the lumbar-pelvic rhythm by sitting, standing, stooping and walking are experienced by the patient, and through strengthening exercises of the 'global muscles' (the 'outer tube system') the local load of the trunk is increased [26-28]. Cardiovascular endurance is trained by a cardio program. The daily activities of the patients are built up, depending on the physical load that the patient can bear.

The lessons aim to modify the patient's cognitions with respect to their complaints, thus reinforcing well behaviours [29]. The group lessons include information on the patient's activities, functional anatomy of the spine, principles of chronic pain, the role and impact of emotions, communication, and finding the balance between the load of daily life and physical capacity.

Individual coaching focuses on the specific needs/problems of the patient. The training is performed in a progressive sequence adjusted to the patient's situation and the clinical experience (estimation) of the therapist. Additional assistance (as required) is provided by a manual therapist, psychologist or therapist specialised in body awareness.

### Prognostic factors

Prognostic factors are assessed at intake and at start of therapy by means of an interview focusing on the patient's history, a physical examination, and on questionnaires. After the 2-month therapy program at SJC, post-treatment follow-up measurements are scheduled at 5 and 12 months after start of therapy. In the present study, the classification into domains as proposed by Pincus et al. (2008), with one additional domain 'Physical characteristics', is used to order the prognostic factors [30].

Table 1 lists the prognostic factors. The prognostic factors include: a) *demographic characteristics *such as educational level, marital status, weight, alcohol, smoking and drug consumption; b) *clinical status *such as body mass index (BMI), pain below the knee, cause and duration of complaints, previous rehabilitation, degree of fatigue [31], low back pain intensity (VAS) [32,33] and disability (QBPDS). [34,35]; c) *psychological characteristics *such as fear avoidance (TSK) [36-42] and quality of life (SF-36) [43]; d) *work-related characteristics *such as employment benefits and work participation in relation to back complaints, and e) *physical characteristics *such as the mobility of lumbar pelvic rhythm (video registration) [44], strength (B-200 isostation) [45,46] and activities of daily living (ADL) consisting of walking, sitting, bicycling and lying. Figure 1 shows the physical tests that are measured at intake, evaluated at the end of therapy, and at 5 months after start of therapy. The reliability and validity of these tests have been established. The Active Straight Leg Raising (ASLR) test [47-49] (0 = not difficult at all, 1 = minimally difficult, 2 = somewhat difficult, 3 = fairly difficult, 4 = very difficult, 5 = unable to do) is positive when the bilateral sum score is ≥ 2 (score range 0-10). The posterior pelvic pain provocation (PPPP) test (0 = no pain, 1 = pain unilateral, 2 = pain bilateral), is positive when the bilateral sum score is ≥ 2 (0-2). For the ligament sacro-iliacale longum dorsal (LDL) test [27] (0 = no pain, 1 = complaint of pain without grimace, flinch, or withdrawal (mild), 2 = pain plus grimace or flinch (moderate), 3 = the examiner is not able to complete the test because of withdrawal (unbearable), the score is positive when the bilateral sum score bilateral is ≥ 2 (score range 0-6). The load transfer adduction test (score best to worse > 129-0 Newton) and abduction (score best to worse > 196-0 Newton) [50] is measured with a microfet in Newtons.

**Table 1:** Baseline characteristics of the study population (n = 1760)

Variables	Population (n = 1760)	Missing value
Number of female participants	1307(74.3)	0
Age in years: M (SD)	40.1(10.6)	0
Weight (kg): M (SD)*	75.3(14.8)	81(4.6)
Height (cm): M (SD)*	172.2(8.8)	70(4.0)

**Demographic factors**		

Low education *	716(40.7)	71(4.0)
Marital status/living with one adult*	1515(86.1)	46(2.6)

**Lifestyle**		

Alcohol consumers; more than 2 per day*	73(4.1)	326(18.5)
Smoking 'yes' *	413(23.5)	326(18.5)
No drug consumers *	1399(79.5)	313(17.8)

**Clinical status**		

Patients with BMI > 25*	783(44.5)	88(5.0)
Duration of complaints in years: M (SD)	7.7(8.8)	0
1 gradual emergence of NLBP	1167(66.3)	30(1.7)
2 sudden emergence of NLBP	563(32.0)	
*Cause*		23(1.3)
1 accident/wrong movement	374(21.3)	
2 after physical overload	73(4.1)	
3 during pregnancy or after delivery	586(33.3)	
4 surgery pelvis/back or after HNP	32(1.8)	
5 unknown	672(38.2)	
Previous revalidation program*	186(10.6)	101(5.7)
Co-morbidity	275(15.6)	88(5.0)
*VAS Pain intensity LBP in mm: M (SD)*		
1 present pain intensity	55.5(23.0)	5(0.3)
2 minimal pain intensity	34.6(21.7)	13(0.7)
3 maximal pain intensity	80.0(16.2)	13(0.7)
*Pain intensity due to CNLBP in the previous 3 months*		52(3.0)
1 stable pain intensity	865(49.1)	
2 increased pain intensity	723(41.1)	
3 decreased pain intensity	120(6.8)	
*VAS degree of fatigue LBP in mm: M (SD)*		
1 present fatigue	56.5(26.6)	118(6.7)
2 minimal fatigue	32.2(23.3)	169(9.6)
3 maximal fatigue	77.8(20.4)	169(9.6)
Disability (QBPDS): M (SD)	51.7(15.6)	8(0.5)

**Psychological factors**		

Fear avoidance (TSK): M (SD)	36.7(7.3)	50(2.8)
*SF-36 (health-related quality of life)*		
PCS	31.8(7.1)	493(28.0)
MCS	46.5(10.3)	493(28.0)

**Work-related factors**		

Employment status benefit	924(52.5)	353(20.1)
*Work participation*		161(9.1)
1 100% working	391(22.2)	
2 1-99% working	488(27.7)	
3 not working	689(39.1)	
4 retired	31(1.8)	
*Less work due to*		460(26.1)
1 complaints	772(43.9)	
2 unemployed	19(1.1)	
3 different reasons	177(10.1)	
4 fully working	332(18.9)	

**Physical examination**		

*LDL positive*		
1 left	1373(78.0)	29(1.6)
2 right	1336(75.9)	31(1.8)
*Mobility (VR) (degrees in flexion): M (SD)*		
1 pelvis in flexion	40.7(15.7)	154(8.8)
2 low back in flexion	47.3(14.3)	154(8.8)
3 pelvis+low back in flexion (ROM)	88.0(24.6)	154(8.8)
*ASLR positive (sum score ≥ 3)*		
1 by general practitioner	1442(81.9)	16(0.9)
2 by patient	1217(69.1)	8(0.5)
*ADL function - duration > 31 min without pain increase*		
1 walking	410(23.3)	10(0.6)
2 cycling	312(17.8)	287(16.3)
3 sitting	432(24.5)	13(0.7)
4 lying	1017(57.8)	15(0.9)
5 standing	106(6.1)	9(0.5)
PPPP positive (uni or bilateral)	1110(63.1)	50(2.8)
Load transfer Abduction (Newton): M (SD)	224.9 (96.4)	137 (7.8)
Load transfer Adduction (Newton): M (SD)	172.5 (87.2)	136 (7.7)
*B200 Isostation (strength) (Newton): M (SD)*		
1 extension	81.6(45.8)	107(6.1)
2 flexion	65.2(45.0)	106(6.0)
3 lateroflexion left	68.1(41.2)	106(6.0)
4 lateroflexion right	74.2(39.4)	106(6.0)
5 rotation left	34.6(23.1)	107(6.1)
6 rotation right	33.4(22.5)	108 (6.1)

Values are numbers (percentages) unless stated otherwise.

* these factors were reported when therapy started, or gathered from the personal status; M = mean; SD = standard deviation; BMI = Body Mass Index; NLBP = non-specific low back pain; VAS = Visual analogue scale; QBPDS = Quebec Back Pain Disability Scale; TSK = Tampa Scale Kinesiophobia; SF-36 = Short Form; PCS = Physical Component Summary; MCS = Mental Component Summary; SCL-90 = Symptom Checklist; GPE = Global Perceived Effect; ADL = activities of daily living; VR = video registration; ASLR = Active Straight Leg Raise; PPPP = Posterior Pelvic Pain Provocation test; LDL = longum dorsal sacroiliac ligament.

The choice to include these specific variables in the analyses as potential prognostic factors is based on a literature review [30], the quality of tests, and clinical experience in the SJC.

### Outcomes

Outcomes are assessed at intake, at the start and end of therapy, and at 5 and 12 months after start of therapy using questionnaires (Figure 1). An international group of back pain researchers recommended a standard battery of outcome measures to represent the multiple dimensions of outcome in the field of back pain [14,16]. We measured improvement of the patient with various measures: 1) pain intensity measured with a visual analogue scale (VAS; at the moment, minimum and maximum) [51,52], 2) low-back-pain-specific disability is measured with the Quebec Back Pain Disability Scale (QBPDS) [53], 3) generic health status. The Short Form (SF-36) is measured at start of therapy [54-58]. The three instruments have shown to be reliable, valid and responsive for a minimal important change (MIC) [32-35,52,53,59-64]. 4) Global Perceived Effect (GPE) of the patient is measured with a 5-point scale (1 = much improved, 2 = slightly improved, 3 = no change, 4 = slightly worsened, 5 = much worsened) [16]. The GPE is proven valid [16,65], and 5) work participation. Work participation is measured by dividing 'current work hours' by 'former work employment hours' prior to CNLBP. No psychometric values are known for this instrument.

### Analyses

Baseline characteristics of the patients are presented as descriptive statistics. Data on the course of CNLPB recovery during treatment are presented in graphs and tables at 5 and 12 months after start of therapy. The development of a multivariate prognostic model is based on principles and methods described by Moons and Altman et al. [66-69]. The relationship between potential prognostic factors and outcome is evaluated using bivariate and multivariate analyses. For all outcome measurements, separate analyses are conducted to investigate prognostic factors at 5 and 12 months after start of therapy. Differences between baseline and follow-up scores are analyzed using repeated measures analysis of variance. Logistic regression is used to determine odds ratios (ORs) of recovery, initially for each variable independently and then in a multiple regression model.

Recovery is operationalised into two definitions: 'improvement in' [16,33,70] and 'absolute' [16,71-73] recovery for each outcome measurement. All analyses are conducted with SPSS for Windows (version 18.0).

## Results

### Baseline Measurements

A total of 2,545 patients [mean age 40.4 (10.9) years; 73.3% women] visited the SJC for an intake consultation between January 2003 and December 2008. Of these, 1,760 patients [mean age 40.1 (10.6) years; 74.3% women] with CNLBP met the inclusion criteria, completed the 2-month therapy program, and were followed up at 5 and 12 months after start of therapy. Of this latter group, 96 followed the 'once a week' therapy program.(with a duration of 4 months). A total of 785 patients [mean age 41.3 (11.5) years; 70.3% women] had the intake consultation but decided not to start therapy: reasons given for this included, only wanting the consultation and/or a diagnosis and/or some advice, referred to another specialist (e.g. psychologist, orthopaedic surgeon), decided not to come, travel distance too far, and unknown reasons.

The distribution of prognostic factors were similar in both the excluded and included groups regarding demographic characteristics, clinical status, psychological status, work-related parameters, and physical examination. Table 1 presents the baseline characteristics of the 1,760 patients; 74.3% is female with a (mean) duration of LBP complaints of 7.8 (SD 8.8) years. Of all patients, 90.2% had stable or increased low back pain intensity in the 3 months prior to intake. Pain intensity and disability showed moderate to severely impaired patients; 43.9% worked less because of their complaints. Of the 1,760 patients, 1,696 (96.4%) completed the 2-month therapy program, 1,564 (88.9%) participated in the 5-month follow-up and 965 (54.8%) completed the 12-month follow-up after start of therapy.

## Discussion

Little information is available on the prognostic factors for recovery in patients with chronic non-specific low back pain. The present study is designed to provide insight into the course and prognostic factors for recovery in patients with CNLBP who are managed in a rehabilitation centre.

The study population was recruited from a multidisciplinary outpatient rehabilitation clinic (part of regular care), which leads to a more pragmatic approach regarding the prognosis of patients with CNLBP. In the 6 years during which patients have been followed for 12 months after start of therapy, the procedure of data recording and the follow-up period has been consistent. This limits information bias for the outcome recovery. Another strength of this study is that use of five domains of recovery allows to describe and analyse a broader perspective of relevant health outcomes for patients with CNLBP.

The study also has some limitations. First, we are unable to present the natural (untreated) course of CNLBP, because all patients receive multidisciplinary treatment during rehabilitation [74,75]. Also, most changes in outcome measurements are reported by the patients themselves, which might lead to some bias. The existing SJC procedure was maintained with regard to the follow-up. This probably decreased the response rate (especially at 12 months after start of therapy) because some patients were no longer motivated or were not approached to provide a response if they did not respond to the postal requests.

### Impact of this study

This study provides information on relevant prognostic factors for recovery, and presents data on the course of patients with CNLBP following a multidisciplinary rehabilitation program.

## Competing interests

The authors declare that they have no competing interests.

## Authors' contributions

All authors participated in the design of the study. KV drafted the manuscript with input from the other authors. All authors read, revised and approved the final manuscript.

## Pre-publication history

The pre-publication history for this paper can be accessed here:

http://www.biomedcentral.com/1471-2474/12/252/prepub
